# Supplementary material for: Tensor networks enable the calculation of turbulence probability distributions
Source: Sci Adv. 2025 Jan 29;11(5):eads5990. doi: 10.1126/sciadv.ads5990 (PMC13109950; doi:10.1126/sciadv.ads5990)
Supplement: 20250129-1 [file sciadv.ads5990.v1.pdf]

PHYSICS

Tensor networks enable the calculation of turbulence probability distributions

Nikita Gourianov[1,2]*, Peyman Givi[1], Dieter Jaksch[2,3], Stephen B. Pope[4]

Copyright © 2025 The Authors, some rights reserved; exclusive licensee American Association for the Advancement of Science. No claim to original U.S. Government Works. Distributed under a Creative Commons Attribution License 4.0 (CC BY).

Predicting the dynamics of turbulent fluids has been an elusive goal for centuries. Even with modern computers, anything beyond the simplest turbulent flows is too chaotic and multiscaled to be directly simulatable. An alternative is to treat turbulence probabilistically, viewing flow properties as random variables distributed according to joint probability density functions (PDFs). Such PDFs are neither chaotic nor multiscale, yet remain challenging to simulate due to their high dimensionality. Here, we overcome the dimensionality problem by encoding turbulence PDFs as highly compressed "tensor networks" (TNs). This enables single CPU core simulations that would otherwise be impractical even with supercomputers: for a 5 + 1 dimensional PDF of a chemically reactive turbulent flow, we achieve reductions in memory and computational costs by factors of $\mathcal{O}(10^6)$ and $\mathcal{O}(10^3)$, respectively, compared to standard finite-difference algorithms. A future path is opened toward something heretofore thought infeasible: directly simulating high-dimensional PDFs of both turbulent flows and other chaotic systems that can usefully be described probabilistically.

INTRODUCTION

Despite the simple and deterministic physical laws governing it, turbulence remains an inherently complex and chaotic phenomenon. It is characterized by large numbers of eddies interacting in intricate and nonlinear ways across wide ranges of spatial and temporal scales, leading to the emergence of chaos. Making matters worse, practically important turbulent flows (e.g., fuel-oxidizer mixtures in combustion) often involve multiple chemically reacting species, which introduces additional nonlinearities and scales. The presence of chaos prohibits predicting the exact dynamics of turbulent flow fields over long periods of time, while the multiscaled nature of the flow fields makes their simulation immensely expensive due to the need to solve sets of coupled partial differential equations (PDEs) on very fine grids.

However, for practical applications it is rarely necessary to know the precise state of a turbulent flow field at every point in space-time. Rather, one is typically more interested in far slower-varying statistical quantities where the fluctuations are averaged out (such as the lift and drag of an aeroplane or the rate of product formation in a chemical process). In the statistical description of turbulence, variables like velocities $\mathbf{U}$, chemical mass-fractions Φ_α, temperatures, etc., are treated as random variables (RVs) distributed according to some one-point, one-time joint probability density function (PDF) (*1*)

$$f = f(\mathbf{u}, \varphi_1, \ldots; \mathbf{x}, t) \tag{1}$$

across space $\mathbf{x}$ and time t, with $\mathbf{u}, \varphi_1$ being sample-space variables corresponding to $\mathbf{U}, \Phi_1$. The trajectory of f completely describes the one-point, one-time statistics of the flow dynamics (*2*), which are the central quantities of interest in practical engineering calculations.

The time evolution of f is modeled by Fokker-Planck PDEs that are straightforward to derive (*3*–*5*) but hard to solve. If f is d-dimensional, assigning M points for each dimension results in a total of M^d gridpoints. Given that d can be as high as $\mathcal{O}(10^3)$ in realistic flows (*6*, *7*), direct schemes like finite differences (FDs) or volumes were long ago dismissed as computationally infeasible (*8*) due to their seemingly exponential cost in d. This spurred the creation of indirect Monte Carlo (MC) algorithms for probabilistic turbulence simulations (*8*). These schemes have proven highly successful, enabling advanced turbulent combustion simulations involving thousands of CPU cores (*9*, *10*). However, the randomness and slow convergence characterizing MC methods can be avoided by directly solving the underlying Fokker-Planck equations.

It is not just probabilistic turbulence calculations that are hindered by the curse of dimensionality: quantum many-body systems are described by states whose sizes also grow exponentially (in the number of particles). However, physically relevant quantum states are known to be highly structured (*11*). Such structure can be exploited to compress the states into approximate, but highly accurate, polynomially large representations known as tensor networks (TNs). TN algorithms allow efficiently evolving these states and analyzing their physical properties without ever leaving the compressed TN representation (*12*–*15*) and have enabled the simulation of otherwise intractable quantum systems like superconductors, ferromagnets, and quantum computers (*16*–*24*). Recently, the TN formalism has begun spreading beyond quantum physics (*25*–*31*).

Decades of empirical experience indicates that f is also highly structured. For instance, in homogeneous turbulence, velocities $\mathbf{U}$ are often distributed normally (*2*), whereas mass fractions Φ_α have been observed to follow normal, exponential, and β distributions in nonreactive flows (*32*). In more complicated reacting flows, the PDFs generally cannot be so simply parameterized (*33*), although they remain smoother and more predictable than the underlying flow fields (*34*).

This work shows that the structure contained in turbulence PDFs is readily exploitable through TNs: using a simple TN known as the "matrix product state" (MPS) ansatz to encode f in a highly compressed format allows us to formulate a scheme for cheaply and directly solving the governing Fokker-Planck equations. When the PDF structure is well-matched to the MPS ansatz, the time-evolution costs just $\sim d \log M$; while standard FD schemes scale as $\sim M^d$. We demonstrate the advantage by looking at the following turbulent flow.

[1]Department of Mechanical Engineering and Materials Science, University of Pittsburgh, Pittsburgh, PA 15261, USA. [2]Clarendon Laboratory, University of Oxford, Oxford OX13PU, UK. [3]Institut für Quantenphysik, Universität Hamburg, Luruper Chaussee 149, 22761 Hamburg, Germany. [4]Sibley School of Mechanical and Aerospace Engineering, Cornell University, Ithaca, NY 14853, USA.
*Corresponding author. Email: nikgourianov@icloud.com

RESULTS

Probabilistic modeling of reactive turbulence

Consider an incompressible, three-dimensional (3D) turbulent flow in which two chemical species are irreversibly reacting: $A + B \rightarrow$ Products. In this system, two chemical species (of mass fractions Φ_1, Φ_2) are stirred by a velocity field $\mathbf{U} = \mathbf{U}(\mathbf{x}, t)$ in 3D space $\mathbf{x}$ across time t. For the sake of simplicity, we here consider only the statistics of the Φ_1, Φ_2 scalar fields by assuming that the large-scale statistical features of the hydrodynamics are known a priori, while modeling the subgrid-scale (SGS), turbulent velocity fluctuations using large eddy simulation (LES), per current best practices (*10*). Doing so eliminates the randomness of $\mathbf{U}$ and reduces the dimensionality to $d = 5 + 1$. Now, $f = f(\varphi_1, \varphi_2; \mathbf{x}, t)$ describes the statistics of the mass fraction fluctuations, which provide the mean mass fractions $\langle\Phi_1\rangle, \langle\Phi_2\rangle$ through

$$\langle\Phi_\alpha\rangle(x,t) = \int_{[0,1]^{\times 2}} \varphi_\alpha f(\varphi_1, \varphi_2; x, t)\,\mathrm{d}\varphi_1 \mathrm{d}\varphi_2 \quad (2)$$

Such PDFs are known as "filtered density functions" (*3*, *35*). Deriving the equation governing f requires SGS closure modeling. Using popular closure models (*35*) gives the Fokker-Planck PDE

$$\frac{\partial f}{\partial t} + \langle U_i \rangle \frac{\partial f}{\partial x_i} - \frac{\partial}{\partial x_i}\left[(\gamma + \gamma_{\mathrm{SGS}})\frac{\partial f}{\partial x_i}\right] = \frac{\partial}{\partial \varphi_\alpha}\left[\Omega_{\mathrm{mix}}(\varphi_\alpha - \langle\Phi_\alpha\rangle) f\right] - \frac{\partial}{\partial \varphi_\alpha}(S_\alpha f) \quad (3)$$

Here, $\mathbf{U}(\mathbf{x}, t)$ is the large-scale (or, "filtered") mean hydrodynamic field across $\mathbf{x} \in [0, l_0]^{\times 3}$, $t \in [0, 2T_0]$, which is set to be a jet flow combined with a Taylor-Green vortex of amplitude $u_0 = l_0 / T_0$ (Materials and Methods, "Flow case definition" section).

The left hand side of Eq. 3 denotes the PDF transport in space and time. The first term is the rate of temporal change, and the second term represents convection by the mean velocity field. The third represents the influence of the molecular (γ) and SGS diffusion [$\gamma_{\mathrm{SGS}}(\mathbf{x}, t)$] coefficients: The former sets the Peclet number $\mathrm{Pe} = u_0 l_0 / \gamma$ and the latter is modeled via the Smagorinsky (*36*) closure $\gamma_{\mathrm{SGS}} = C_s \frac{\Delta_\ell^2}{2} \sqrt{\sum_{ij}\left(\frac{\partial U_i}{\partial x_j} + \frac{\partial U_j}{\partial x_i}\right)^2}$, with C_s an empirical constant and Δ_ℓ the LES filter width (both are specified in Materials and Methods, "Flow case definition" section).

The right hand side of Eq. 3 designates transport in the composition space ["composition" since the $\varphi_1, \varphi_2 \in [0,1]^{\times 2}$ mass fractions define the composition of the fluid]. The first term represents scalar mixing from the SGS turbulence and is modeled via the popular least mean square estimation (LMSE) (*37*) closure $\Omega_{\mathrm{mix}} = C_\Omega \frac{\gamma + \gamma_{\mathrm{SGS}}}{\Delta_\ell^2}$, with C_Ω the SGS mixing rate. The final term denotes the effects of chemical reaction. For the binary reaction scheme considered here, $S_1 = S_2 = -C_r \varphi_1 \varphi_2$, where C_r denotes the reaction rate that defines the Damköhler number $\mathrm{Da} = C_r l_0 / u_0$.

To solve Eq. 3, we discretize f at every point in time on a $M = 128$, $d = 5$ Cartesian grid, but parameterize it as an MPS-network using far fewer variables than the 128^5 gridpoints resolving it. This allows us to use a simple Runge-Kutta 2, FD scheme (Materials and Methods, "FD discretization" section) to solve Eq. 3 and time evolve the MPS-PDF.

An initial MPS simulation (Materials and Methods, "MPS algorithm" section) is performed in Fig. 1 of a purely mixing flow without chemical reactions ($\mathrm{Da} = 0$). The PDF is illustrated at two points in $\mathbf{x}$ along with the scalar-ratio $\langle\Phi_1\rangle / \langle\Phi_2\rangle$ at four different times, showing how the initially orderly, unmixed flow state is driven toward a fully-mixed $\langle\Phi_1\rangle / \langle\Phi_2\rangle \approx 1$ state by SGS and large-scale convective and diffusive mixing. The SGS mixing leads to the PDF concentrating, while the diffusion and mean-flow convection induces multimodality in the PDF. The MPS simulation is highly accurate (Fig. 2B), yet the number of variables parameterizing the PDF (NVPP) is only $\mathcal{O}(1/10^5)$ of an equivalent, classically implemented FD scheme [Materials and Methods, Eq. 12].

MPS encoding

In our MPS encoding, the discretized, high-dimensional $f(\varphi_1, \varphi_2, x_1, x_2, x_3)$ is decomposed into a 1D chain of tensors, where the $\varphi_1, \varphi_2, x_1, x_2, x_3$ dimensions are sequentially mapped to tensors from left to right, with each dimension itself decomposed into multiple tensors lengthscale by lengthscale {analogously to the "sequential, serial" ordering in [(*27*), Fig. 1] and [(*38*), Eq. 9]}. This encoding exploits two separate structures that characterize the solution of Eq. 3: First, the general smoothness of turbulence PDFs; second, that the different dimensions of $f(\varphi_1, \varphi_2; \mathbf{x}, t)$ are unlikely to be strongly coupled at low C_Ω, because for $C_\Omega = 0$ the PDF is separable (Supplementary Text, "Separability of Fokker-Planck equation" section).

Matching the structure of the PDF in this way allows for an MPS encoding that is both accurate and parsimonious. The MPS representation (like any TN) can be systematically compressed, i.e., the NVPP reduced, by varying a hyperparameter known as the maximum bond dimension χ. This hyperparameter regulates the maximal size of the "bonds" between the tensors, which is equivalent to the maximum allowed coupling between the tensors and, in turn, between the different lengthscales and dimensions of f. For example, setting $\chi = 1$ forbids any coupling between the tensors and makes the NVPP minimal, while picking χ sufficiently large makes the MPS representation exact and $\mathrm{NVPP} = M^5$ like in the standard representation. Setting χ to be small in turn leads to a low NVPP, but the MPS encoding will still remain accurate if it reflects the structure of f sufficiently well.

Validation of algorithm

We now investigate how well the MPS parameterization fits the solution of Eq. 3 in practice. To determine the χ required to accurately simulate the dynamics of the RVs Φ_1, Φ_2 underlying the PDF, the composition space transport parameters C_Ω, Da are varied, while fixing the hydrodynamic variables $\langle \mathbf{U} \rangle$, γ_{SGS}, and Pe to those used in Fig. 1.

Increasing C_Ω is expected to lead to higher coupling between the different dimensions of f, which reduces the efficiency of our MPS encoding, i.e., increasing C_Ω requires an increased χ to maintain accuracy. To verify, we first set $\mathrm{Da} = 0$ because this allows us to accurately compute $\langle\Phi_\alpha\rangle$ independently of Eq. 3 (see Materials and Methods, "Moment equations" section) and benchmark the accuracy of the computed MPS-PDF across C_Ω, χ. The benchmark is shown in Fig. 2 (A and B). The $\langle\Phi_1\rangle / \langle\Phi_2\rangle$ ratios in Fig. 2A depict how the MPS-PDF means approach their numerically exact equivalent when χ increases and C_Ω decreases. All the cases, including the ones with lowest accuracy, correctly trend toward a fully mixed equilibrium state where $\langle\Phi_1\rangle / \langle\Phi_2\rangle \approx 1$. Figure 2B quantitatively shows that the root mean square error (RMSE) in terms of both the Reynolds-averaged mean mass fraction

$$\overline{\langle\Phi_\alpha\rangle} = \int_{[0,l_0]^{\times 3}} \langle\Phi_\alpha\rangle\,\mathrm{d}x \quad (4)$$

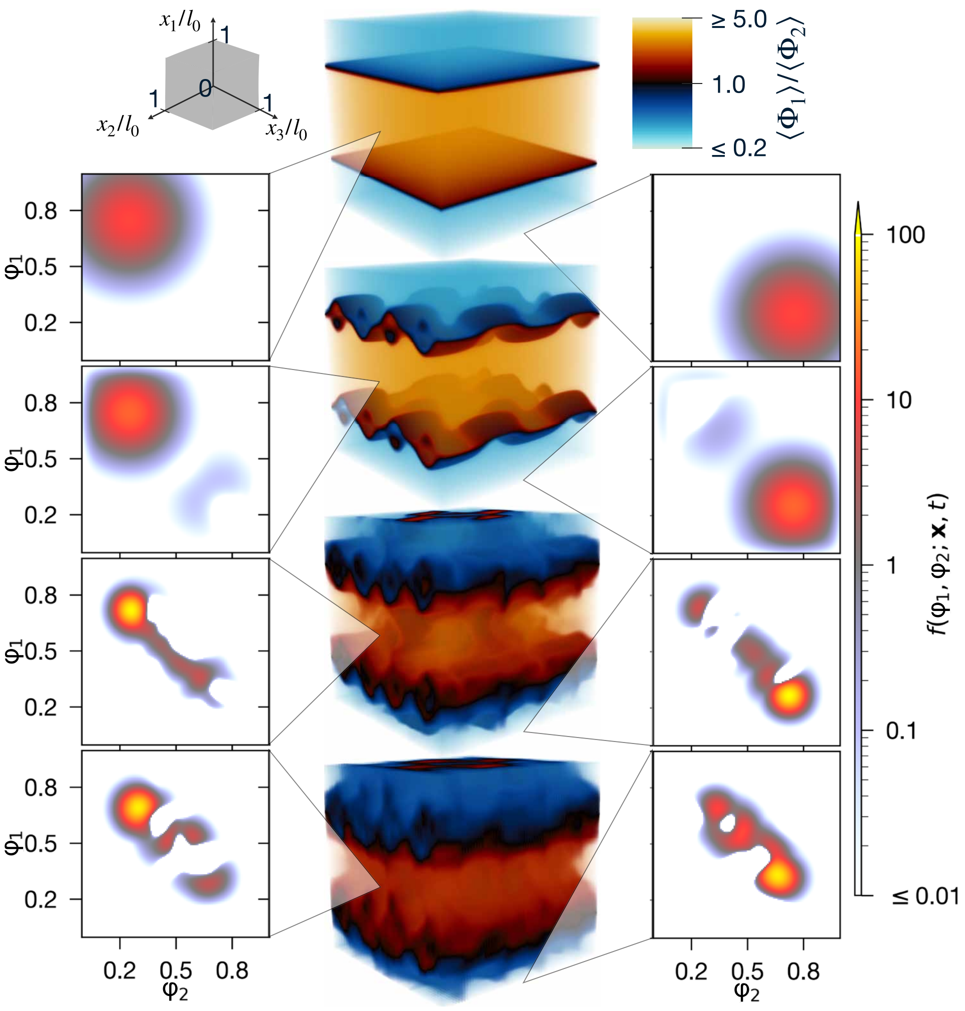

Fig. 1. High-dimensional PDF of a flow undergoing turbulent mixing revealed by TN simulation. Here, the Fokker-Planck Eq. 3 is solved for a PDF $f(\varphi_1, \varphi_2; \mathbf{x}, t)$ over chemical mass fractions φ_1, φ_2, at $C_\Omega = 1$, Da $= 0$ in the presence of a Pe $= 10^3$ velocity field $\mathbf{U}$ characterized by vortices and a jet along x_1 (Materials and Methods, "Flow case definition" section). The 5D $f(\varphi_1, \varphi_2; \mathbf{x}, t)$ is represented by a MPS ansatz at $\chi = 128$, on a $128^{\times 5}$ grid and is visualized here for $\mathbf{x}/l_0 = \left(\frac{1}{2}, 1, \frac{1}{2}\right)$ and $\mathbf{x}/l_0 = \left(0, \frac{1}{2}, 1\right)$ at times $t/T_0 = 0, 0.125, 1, 2$ in the left and right columns, while corresponding mean mass fraction ratios $\langle\Phi_1\rangle / \langle\Phi_2\rangle$ are shown in the center.

and $\langle\Phi_\alpha\rangle$ decrease roughly polynomially in χ for all C_Ω.

Figure 2C depicts how varying the Damköhler number affects the accuracy of the MPS algorithm. When Da > 0, any moments $\langle\Phi_\alpha^n\rangle, n \in \mathbb{Z}_{\geq 0}$ higher than the norm $\langle\Phi_\alpha^0\rangle = \langle 1\rangle$ can no longer be independently computed. We therefore rather look at two quantities that our simulation must preserve: the norm, which must equal unity across $\alpha, \mathbf{x}, t$, and the difference in consumption between the two species $\overline{\langle\Phi_1\rangle - \langle\Phi_2\rangle}$, which should be zero for all t due to the symmetry of S_α and the initial conditions. The figure indicates these two quantities becoming increasingly preserved when, again, C_Ω decreases and χ increases. Notably, the errors decrease roughly polynomially in χ. However, varying Da has little impact on the accuracy. This is because the chemical reaction largely just drives the PDF in compositional space toward the origin (as seen in Fig. 3), without significantly affecting its structure.

Computational complexity

The maximal bond dimension χ not only sets the accuracy of the MPS simulation but also determines the computational cost. Because our MPS algorithm (Materials and Methods, "MPS algorithm" section) implements a finite difference method within the MPS framework, it must perform the MPS equivalent of operations like element-wise, matrix-matrix and matrix-vector multiplications, matrix and vector additions and subtractions, and inner and outer products, in addition to MPS-specific operations like singular values and QR decompositions to enforce the maximal bond dimension and ensure the MPS stays in the numerically manageable "canonical form" (*14*, *15*). It is straightforward to show (*39*) that these MPS operations all cost $O(\chi^q d \log M)$ asymptotically, with $q \in \mathbb{Z}_{>0}$ depending on the operation.

The element-wise multiplication operation is the most expensive at $q = 4$ [(*39*), section 4.6], making the asymptotic complexity of our scheme as a whole $O(\chi^4 d \log M)$ per timestep. Thus, for M_t timesteps, the total cost of the time evolution will approach $O(M_t \chi^4 d \log M)$ at very large χ; although in practice for small and intermediate χ, the empirical cost scales much milder (Supplementary Text, "Empirical computational cost" section). In comparison, standard FD schemes are exponentially more expensive in d, costing $O(M_t M^d)$.

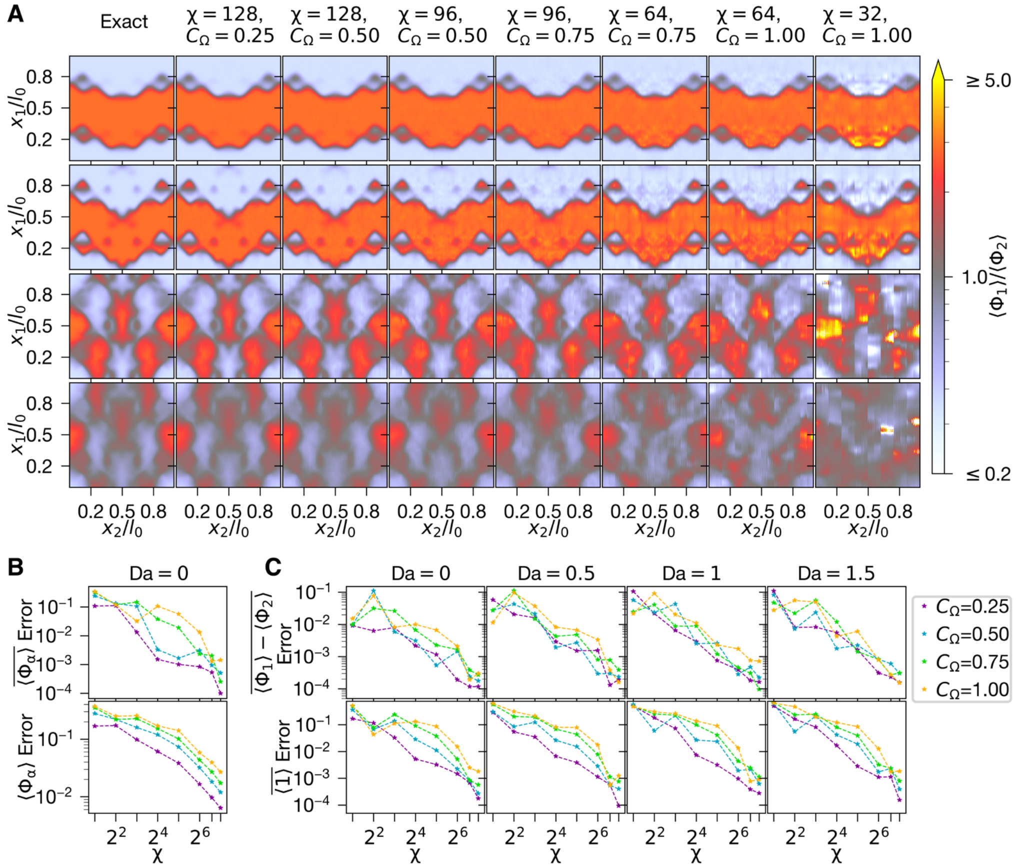

Fig. 2. Accuracy convergence of TN algorithm. The influence of χ, C_Ω and Da on the accuracy of the MPS simulation is outlined here. (**A**) and (**B**) contrast numerically exact means against those extracted from the MPS algorithm. In (A), the ratio $\langle\Phi_1\rangle/\langle\Phi_2\rangle$ is visualized at $x_3\,/\,l_0\,=\,\frac{1}{2}$ for times $t\,/\,T_0$ = 0.125, 0.25, 1, and 2, top to bottom. The leftmost column corresponds to the exact solution (which can only be practically computed for Da = 0 through Eq. 9), while the next six columns come from MPS simulations at varying χ, C_Ω. The differences between the exact and MPS solutions are quantified in the lower (B) plot; the upper (B) plot shows how well the total species amounts $\overline{\langle\Phi_\alpha\rangle}$ (see Eq. 4) are preserved through the simulation. In (**C**), the RMSE in two basic statistics is computed: the difference in species consumption $\overline{\langle\Phi_1\rangle-\langle\Phi_2\rangle}$, which should always equal zero, and the space-averaged norm $\overline{\langle 1\rangle}$, which should always equal one. All RMSEs are mathematically defined in Materials and Methods, "Error measures" section.

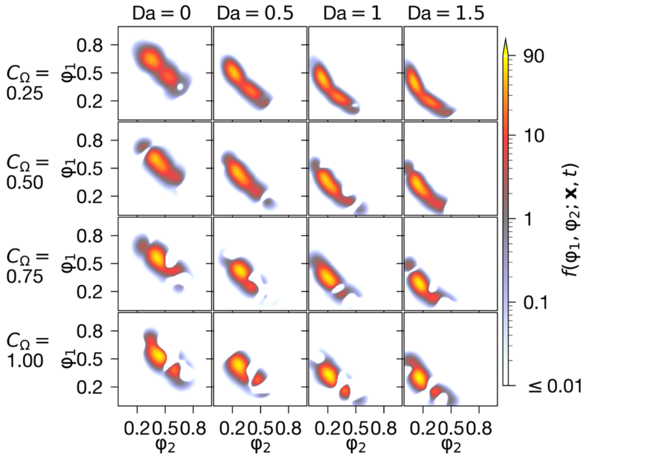

Fig. 3. Final PDF for various flow parameters. The PDFs at the end of the simulation $(t/T_0=2)$ are shown here in the center of the spatial domain $\left[\mathbf{x}/l_0=\left(\frac{1}{2},\frac{1}{2},\frac{1}{2}\right)\right]$ for all combinations of C_Ω, Da. The PDFs are computed using χ = 128 MPS simulations.

There is also the question of preparing initial states and extracting statistics. Regarding the former, the 3D $\langle \mathbf{U}\rangle(\mathbf{x}), \gamma_{SGS}(\mathbf{x})$ and d-dimensional $f\left(\varphi_1,\varphi_2;\mathbf{x},t=0\right)$ can be computed using either the prolongation method {see [(*39*), section 4.4] and (*40*)} or the tensor-cross algorithm (*30*, *41*–*44*), both at $O\left(\chi^3 d\log M\right)$ cost. As for the latter, computing expectation values boils down to doing the MPS equivalent of matrix-vector multiplication and inner products, which are, as noted previously, inexpensive and straightforward operations. For instance, at any given timestep, the 3D mean $\langle\Phi_\alpha\rangle$ can be extracted from f at $O\left(M^3\chi^2 d\log M\right)$ complexity, while the cost is $O\left(\chi^2 d\log M\right)$ for the scalar $\overline{\langle\Phi_\alpha\rangle}$.

Integrated quantities

The satisfactory accuracy and subexponential cost of our MPS scheme allows us to directly compute the PDF, visualize it, and extract from it all relevant integrated quantities.

Figure 3 shows the influence of mixing and chemical reactions on the PDF. As expected, in the absence of chemical reaction, both species tend toward the fully mixed values $\langle\Phi_\alpha\rangle(t\to\infty)\to 0.5$ at a rate governed by C_Ω. Whereas in the reacting flow simulations,

$\langle\Phi_\alpha\rangle(t\to\infty)\to 0$ at a rate that increases with C_Ω and Da. Visually, we see that increasing C_Ω leads to a PDF that is more concentrated along $\varphi_1=\varphi_2$ (implying a more mixed fluid), while increasing Da takes the PDF closer to the origin (meaning more of the reactants have been consumed). Multimodality is also evident in some PDFs; this is a result of convective and diffusive transport in $\mathbf{x}$-space.

The trends of Fig. 3 are reflected in the integrated quantities plotted in Fig. 4. The first row illustrates $\overline{\langle\Phi_1\rangle}$ going from being conserved at Da = 0 to being consumed at rates increasing with Da, as expected. The consumption also slightly increases with the SGS mixing rate. The following row shows the negative of the (Reynolds averaged) scalar covariance

$$R_{12}=\overline{\langle\Phi_1\rangle\langle\Phi_2\rangle}-\overline{\langle\Phi_1\rangle}\;\overline{\langle\Phi_2\rangle} \tag{5}$$

which decays in t due to $\langle\Phi_1\rangle/\langle\Phi_2\rangle$ approaching unity as the flow becomes increasingly mixed. Last, the last row exhibits the covariance

$$Y_{12}=\overline{\langle\Phi_1\rangle\langle\Phi_2\rangle}-\overline{\langle\Phi_1\rangle}\;\overline{\langle\Phi_2\rangle} \tag{6}$$

At initial times, $-\overline{Y_{12}}$ increases due to large gradients in $\langle\Phi_\alpha\rangle$, followed by a decrease due to mixing (with higher mixing-rates leading to a faster decay). The ratio $R_{12}/\left(R_{12}+\overline{Y_{12}}\right)$ is consistently above one half, implying that most of the energy of the eddies is resolved during the simulations. The statistical trends observed in Fig. 4 are consistent with those reported in turbulence literature (*35*).

DISCUSSION

The results imply that MPSs are able to efficiently exploit structure within turbulence PDFs. The PDF $f\left(\varphi_1,\varphi_2;\mathbf{x},t\right)$ of our 3D chemically reactive flow case [Eq. 3] is of an orderly shape, and the coupling between its dimensions is limited by the SGS mixing rate C_Ω. Exploiting these structures permits our MPS scheme to accurately and efficiently represent f and evolve it through time. In the future, a more realistic model should be considered where the velocity field is also included within the PDF, turning $f=f\left(\mathbf{u},\varphi_1,\varphi_2;\mathbf{x},t\right)$ into a $d=8+1$ dimensional object.

Ensuring the MPS algorithm maintains both accuracy and efficiency requires carefully selecting χ (see figs. S1 to S3). Figure 2 indicates that varying the Damköhler number does not significantly affect the accuracy, while increasing the SGS mixing rate requires χ to in turn increase as $\chi\sim\mathrm{poly}\left(C_\Omega\right)$ for accuracy to be maintained. Setting

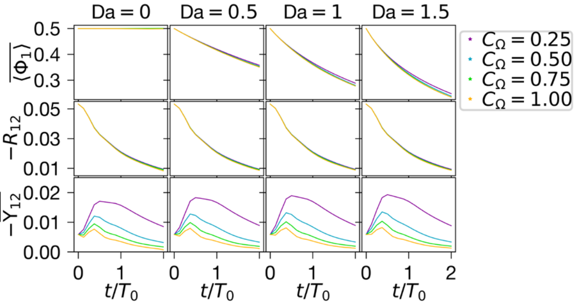

Fig. 4. Statistics extracted from PDF for different flow parameters. In the first row, the total amount of the first species is plotted. The next row is of the Reynolds-averaged covariance R_{12}, while the final row quantifies the space-averaged covariance $\overline{Y_{12}}$. These quantities are defined in Eqs. 4 to 6. The PDFs are computed at $\chi = 128$.

χ excessively high is expensive due to the $O\left(\chi^4 d\log M\right)$ asymptotic cost of the algorithm. However, for slower mixing rates, high accuracy is achievable at very low χ even when the chemical reaction rates are high. For instance, at $C_\Omega=0.25$, Da = 1.5, the algorithm is accurate with just $\chi=32$. This is equivalent to respective $\mathcal{O}\left(10^6\right)$ and $\mathcal{O}\left(10^3\right)$ factor reductions in memory and computational costs [Materials and Methods, Eq. 12, and Supplementary Text, "Empirical computational cost" section] compared to conventional FD schemes, allowing the time evolution to be executed on a single CPU core in only a couple of hours, instead of days on a supercomputer.

The results shown here are only an early indication of what is possible: there exists great scope for improvement in both the algorithm and its implementation. For example, using tensor-cross or other algorithms (*45*) to perform element-wise multiplications might reduce the complexity of our scheme to $\sim\chi^3$ without significantly sacrificing accuracy. Furthermore, better optimized software running on specialized computing architectures will allow for much larger bond dimensions and system sizes: We are currently simulating a $M^d=128^5=2^{35}$ grid at $\chi=128$, while the current record is a quantum physics simulation on a grid equivalent of $M^d=2^{400}$ at $\chi=32768$, performed on a tensor processing unit pod (*46*).

We decided to use an MPS ansatz because it closely matches the structure of the PDF for this particular flow; other flows may have different PDFs for which alternative ansatze could be better suited (*47*). Fortunately, there exists a rich and growing selection of TNs to pick from, each carrying their advantages and disadvantages. These range from 2D generalizations of MPSs (*48*), hierarchical networks (*49*, *50*), and even networks that might someday leverage quantum hardware (*51*). There is also the exciting prospect of catering the TN ansatz to the structure of the PDF in an automated manner (*52*). Typically, more complex ansatze are able to encode solutions with higher accuracy at lower χ but are costlier to manipulate. Balancing such considerations while exploring alternative TN geometries for probabilistic turbulence simulations is a promising avenue of future investigation.

Turbulence is just one example of a complex system; there are many others, ranging from biological organisms to financial markets (*53*). These kind of systems exhibit chaotic and unpredictable dynamics that ultimately require statistical descriptions (*54*). The most fundamental way of doing so is by modeling their PDFs. Yet, such PDFs are typically prohibitively high dimensional (as displayed here for the case of turbulence), which has made solving their governing Fokker-Planck equations infeasible, until now. This work is a first demonstration in how the problem can be overcome via a simple TN. More advanced TN ansatze and algorithms will be developed in time, holding the promise of enabling large-scale probabilistic simulations both within the field of fluid dynamics and beyond.

MATERIALS AND METHODS

Flow case definition

In Eq. 3, the mean velocity field $\langle\mathbf{U}\rangle$ is set a priori. To ensure adequate convective mixing and for the flow to be interesting, we elected to set the velocity field to a jet moving through a Taylor-Green vortex

$$\begin{aligned}\langle U_1\rangle/u_0 &= \cos kx_1\sin kx_2\sin kx_3 - e^{-\frac{(x_2/l_0-1/2)^2+(x_3/l_0-1/2)^2}{2(1/6)^2}}\\ \langle U_2\rangle/u_0 &= \sin kx_1\cos kx_2\sin kx_3,\\ \langle U_3\rangle/u_0 &= -2\sin kx_1\sin kx_2\cos kx_3\end{aligned} \tag{7}$$

Here, the vortex wavenumber k is set to $k = 4\pi / l_0$.

The initial ($t = 0$) PDF is chosen to be a Gaussian step function

$$f(t=0) = \frac{1}{2\pi(1/8)^2} \begin{cases} e^{-\frac{(\varphi_1-3/4)^2+(\varphi_2-1/4)^2}{2(1/8)^2}}, & \frac{1}{4} \le x_1 < \frac{3}{4}, \\ e^{-\frac{(\varphi_1-1/4)^2+(\varphi_2-3/4)^2}{2(1/8)^2}}, & \text{otherwise} \end{cases} \tag{8}$$

that has undergone numerical smoothing in the $\mathbf{x}$ dimensions (the smoothing is meant to soften the step function at the $x_1 = \frac{1}{4}, \frac{3}{4}$ boundary sufficiently to avoid numerical instabilities during time evolution). The initial PDF is illustrated in the first rows of Fig. 1.

The $M = 128$, $d = 5$ grid is sufficient for the simulation to be conducted with parameters that make physical sense: we set $C_s = 0.11$, $\Delta_\ell = 3\Delta x = 3l_0 / M$, $\mathrm{Pe} = 10^3$, $C_\Omega \in [0.25,1]$, and $\mathrm{Da} \in [0,1.5]$.

Moment equations

The zeroth moment of the Fokker-Planck Eq. 3 recovers the hydrodynamic continuity equation $\nabla \cdot \langle \mathbf{U} \rangle = 0$, while the first gives an equation for the mean mass fractions $\langle \Phi_\alpha \rangle (\mathbf{x}, t)$

$$\frac{\partial \langle \Phi_\alpha \rangle}{\partial t} + \langle U_i \rangle \frac{\langle \Phi_\alpha \rangle}{\partial x_i} = \frac{\partial}{\partial x_i} \left[(\gamma + \gamma_{\mathrm{SGS}}) \frac{\partial \langle \Phi_\alpha \rangle}{\partial x_i} \right] + \langle S_\alpha \rangle \tag{9}$$

In nonreactive flows ($\mathrm{Da} = 0$), $S = 0$ and Eq. 9 can be cheaply and accurately solved using a standard FD scheme to obtain a "numerically exact" $\langle \Phi_\alpha \rangle$ solution (in the sense that there is no truncation error in χ, as explained in Materials and Methods, "Error measures" section). This is used to check the accuracy of the MPS algorithm in Fig. 2 (A and B). It is not possible to obtain a numerically exact $\langle \Phi_\alpha \rangle$ when $\mathrm{Da} > 0$, because a closure model would be required for $\langle S_\alpha \rangle$.

FD discretization

The simulations are performed on equidistant Cartesian grids with $M = 128$ gridpoints along each dimension. The derivatives in Eqs. 3 and 9 are discretized in a simple manner: the temporal derivative with an explicit Runge-Kutta 2 scheme and a second-order-accurate central FDs (CFD2) discretization of the $\mathbf{x}, \varphi_1, \varphi_2$ derivatives.

However, discretizing Eq. (3) creates the practical challenge of handling delta-functions. The LMSE model forces each Φ_α toward $\langle \Phi_\alpha \rangle$ at every $\mathbf{x}, t$, equivalent to the PDF in composition space moving toward a delta function centered around the mean of the mass fractions. Resolving delta functions on discretized grids is difficult, as their sharp gradients reduce the accuracy and stability of any numerical scheme used to compute the PDF transport. While often this is dealt with by using highly dissipative discretizations of derivatives (e.g., upwinding), we rather choose to simply modify the LMSE model in Eq. 3 through the addition of an artificial dissipation term to the compositional space. Doing this while discretizing the Fokker-Planck PDE results in

$$\frac{\Delta f}{\Delta t} + \langle U_i \rangle \frac{\Delta f}{\Delta x_i} - \frac{\Delta}{\Delta x_i} \left[(\gamma + \gamma_{\mathrm{SGS}}) \frac{\Delta f}{\Delta x_i} \right] = \frac{\Delta}{\Delta \varphi_\alpha} \left[\Omega_{\mathrm{mix}} (\varphi_\alpha - \langle \Phi_\alpha \rangle) f + C_\Omega \mu \frac{\Delta f}{\Delta \varphi_\alpha} \right] - \frac{\Delta}{\Delta \varphi_\alpha} (S_\alpha f) \tag{10}$$

with the artificial dissipation governed by μ. This parameter needs to be set to be as small as possible to minimally affect the accuracy, while still being large enough to ensure f is well resolved on M. From trial and error, we find $\mu = 4 \cdot 10^{-3} \frac{u_0}{l_0}$ works well for $M = 128$.

Particular care must be taken when defining the boundary conditions for this problem. While in $\mathbf{x}$-space, one may simply assume periodic boundaries, in compositional space, the boundary conditions must be defined in a way that stops probability leaking out of the domain. This is achieved by making the composition space ghosts points for any order-n discrete derivative of f follow

$$\sum_{i=0}^{M-1} \left[\frac{\Delta^n f}{\Delta \varphi_\alpha^n} \right]_i = 0 \tag{11}$$

with i denoting a discretized (equidistantly distributed) lattice point. Equation 11 imposes $f_{-1} = -f_0$ & $f_M = -f_{M-1}$ for the first derivative, and $f_{-1} = f_0$ & $f_M = f_{M-1}$ for the second, under our CFD2 discretization.

MPS algorithm

Our MPS algorithm implements the aforementioned RK2-CFD2 scheme on the MPS manifold (*14*, *55*). This entails parameterizing all the vectors (like f, $\langle U_i \rangle$, and $\langle \Phi\alpha \rangle$) in Eq. 10 as MPSs [(*39*), section 3.4], and the matrices (e.g., $\Delta / \Delta x_i$) as analogous matrix product operators [(*39*), section 3.5]. Then, within the MPS format, the time-stepping is performed in a standard manner using the arithmetic operations outlined in the "Computational complexity" section.

It is essential to control the bond dimension during the MPS simulation. The arithmetic operations that time-evolve f lead to its bond dimension growing exponentially in time, if not truncated [(*39*), section 3.5.3]. In our code, we use the singular values decomposition to truncate the bond dimension of f such that it is always limited to χ. As for the other vectors and matrices, these objects remain constant in time and their bond dimensions are all of order $\mathcal{O}(10)$.

The maximal bond dimension χ defines the NVPP. For an MPS representation of f, the number of parameters becomes

$$\mathrm{NVPP} = 2 \sum_{n=1}^{N} p(n-1) p(n) - \sum_{n=1}^{N-1} p(n)^2 \tag{12}$$

with $N = \log_2 M^d$, (M must be a power of 2) being the number of tensors in the MPS, and $p(n) = \min(2^n, 2^{N-n}, \chi)$ being the size of the nth bond of the MPS. The first sum gives the total number of parameters in the MPS, while the second sum represents the intrinsic gauge degrees of freedom of the MPS format (*55*). When χ is maximal, i.e., $\chi = 2^{\lfloor N/2 \rfloor}$, we get $\mathrm{NVPP} = 2^N = M^d$ and that f is represented exactly on the $M^{\times d}$ grid.

Error measures

The errors in Fig. 2 (B and C) are computed using the RMSE measure across χ. In the first figure, the upper $\mathrm{Error}_{2b\uparrow}$ and lower $\mathrm{Error}_{2b\downarrow}$ are computed by averaging the spatially averaged mean quantities across α, t and $\alpha, t, \mathbf{x}$, respectively. In Fig. 2C, the averaging is done across just t and $t, \mathbf{x}$ to compute $\mathrm{Error}_{2c\uparrow}$, $\mathrm{Error}_{2c\downarrow}$. Mathematically, these errors can be expressed as

$$\begin{aligned} \mathrm{Error}_{2b\uparrow}(\chi) &= \sqrt{\frac{1}{2} \sum_{\alpha=1,2} \mathscr{E}_t \left[\overline{\langle \Phi_\alpha \rangle}(\chi), 1/2 \right]}, \\ \mathrm{Error}_{2b\downarrow}(\chi) &= \sqrt{\frac{1}{2} \sum_{\alpha=1,2} \mathscr{E}_{t,x} \left[\langle \Phi_\alpha \rangle(\chi), \langle \Phi_\alpha \rangle(\text{exact}) \right]} \\ \mathrm{Error}_{2c\uparrow}(\chi) &= \sqrt{\mathscr{E}_t \left[\overline{\langle \Phi_1 \rangle}(\chi) - \overline{\langle \Phi_2 \rangle}(\chi), 0 \right]}, \\ \mathrm{Error}_{2c\downarrow}(\chi) &= \sqrt{\mathscr{E}_{t,x} \left[\langle 1 \rangle(\chi), 1 \right]} \end{aligned} \tag{13}$$

with $\langle \Phi_\alpha \rangle (\chi)$ being extracted from the MPS-PDF solution of Eq. 10 while $\langle \Phi_\alpha \rangle$ (exact) is the solution found by directly solving Eq. 9 with a standard RK2-CFD2 FD scheme; this solution is "numerically exact" in the sense that it does not suffer from any truncation error in χ [although a truncation error from the FD discretization itself remains, this error is slight due to the smoothness of $\langle \Phi_\alpha \rangle$ (exact)]. The functions

$$\mathrm{E}_{t,\mathbf{x}}\left(g, g_0\right) = \int_{[0,l_0]^{\times 3}} \frac{\mathrm{d}\mathbf{x}}{l_0^3} \mathrm{E}_t\left[g(\mathbf{x}), g_0(\mathbf{x})\right],$$
$$\mathrm{E}_t\left(g, g_0\right) = \int_0^{2T_0} \frac{\mathrm{d}t}{2T_0}\left[g(t) - g_0(t)\right]^2 \qquad (14)$$

implement temporal (E_t) and space-time ($\mathrm{E}_{t,\mathbf{x}}$) averaging.

Note that since both time and space are discretized during the simulations, the above integrals are both performed numerically using a simple step quadrature. In space, the integrals are computed using all the $M = 128$ gridpoints along each dimension. In time, the integral is computed over the 17 time samples $t = 0, \frac{T_0}{8}, \frac{T_0}{4}, \frac{3T_0}{8}, \ldots, 2T_0$.

Supplementary Materials

This PDF file includes:
Supplementary Text
Figs. S1 to S3
References

REFERENCES AND NOTES

1. E. Hopf, Statistical hydromechanics and functional calculus. *J. Rat. Mech. Anal.* **1**, 87–123 (1952).
2. A. S. Monin, A. M. Yaglom, *Statistical Fluid Mechanics* (MIT Press, Cambridge, MA) (1975).
3. S. B. Pope, *Turbulent Flows* (Cambridge Univ. Press, Cambridge, UK) (2012).
4. R. O. Fox, *Computational Models for Turbulent Reacting Flows* (Cambridge Univ. Press, Cambridge, UK) (2009).
5. C. Dopazo, Recent Developments in PDF Methods, in *Turbulent Reacting Flows*, P. A. Libby, F. A. Williams, Eds. (Academic Press, London, England), chap. 7, pp. 375–474 (1994).
6. F. A. Williams, *Combustion Theory* (The Benjamin/Cummings Publishing Company, Menlo Park, CA), Ed. 2. (1985).
7. D. Livescu, A. G. Nouri, F. Battaglia, P. Givi, Eds. *Modeling and Simulation of Turbulent Mixing and Reaction: For Power, Energy and Flight* (Springer, Germany) (2020).
8. S. B. Pope, PDF methods for turbulent reactive flows. *Prog. Energy Combust. Sci.* **11**, 119–192 (1985).
9. V. Hiremath, S. R. Lantz, H. Wang, S. B. Pope, Computationally-efficient and scalable parallel implementation of chemistry in simulations of turbulent combustion. *Combust. Flame* **159**, 3096–3109 (2012).
10. H. Zhou, P. Givi, Z. Ren, Filtered density function: A stochastic closure for coarse grained simulation, in *Coarse Graining Turbulence: Modeling and Data-Driven Approaches and their Applications* (Cambridge Univ. Press), chap. 4 (2025).
11. D. Poulin, A. Qarry, R. Somma, F. Verstraete, Quantum simulation of time-dependent Hamiltonians and the convenient illusion of Hilbert space. *Phys. Rev. Lett.* **106**, 170501 (2011).
12. S. R. White, Density matrix formulation for quantum renormalization groups. *Phys. Rev. Lett.* **69**, 2863–2866 (1992).
13. G. Vidal, Efficient classical simulation of slightly entangled quantum computations. *Phys. Rev. Lett.* **91**, 147902 (2003).
14. U. Schollwöck, The density-matrix renormalization group in the age of matrix product states. *Ann. Phys.* **326**, 96–192 (2011).
15. R. Orús, Tensor networks for complex quantum systems. *Nat. Rev. Phys.* **1**, 538–550 (2019).
16. S. R. Clark, D. Jaksch, Dynamics of the superfluid to Mott-insulator transition in one dimension. *Phys. Rev. A* **70**, 043612 (2004).
17. A. Feiguin, S. Trebst, A. W. W. Ludwig, M. Troyer, A. Kitaev, Z. Wang, M. H. Freedman, Interacting anyons in topological quantum liquids: The golden chain. *Phys. Rev. Lett.* **98**, 160409 (2007).
18. M. Cheneau, P. Barmettler, D. Poletti, M. Endres, P. Schauß, T. Fukuhara, C. Gross, I. Bloch, C. Kollath, S. Kuhr, Light-cone-like spreading of correlations in a quantum many-body system. *Nature* **481**, 484–487 (2012).
19. S. Trotzky, Y.-A. Chen, A. Flesch, I. P. McCulloch, U. Schollwöck, J. Eisert, I. Bloch, Probing the relaxation towards equilibrium in an isolated strongly correlated one-dimensional Bose gas. *Nat. Phys.* **8**, 325–330 (2012).
20. B.-X. Zheng, C.-M. Chung, P. Corboz, G. Ehlers, M.-P. Qin, R. M. Noack, H. Shi, S. R. White, S. Zhang, G. K.-L. Chan, Stripe order in the underdoped region of the two-dimensional Hubbard model. *Science* **358**, 1155–1160 (2017).
21. C. Huang, F. Zhang, M. Newman, X. Ni, D. Ding, J. Cai, X. Gao, T. Wang, F. Wu, G. Zhang, H.-S. Ku, Z. Tian, J. Wu, H. Xu, H. Yu, B. Yuan, M. Szegedy, Y. Shi, H.-H. Zhao, C. Deng, J. Chen, Efficient parallelization of tensor network contraction for simulating quantum computation. *Nat. Comput. Sci.* **1**, 578–587 (2021).
22. Y. Zhou, E. M. Stoudenmire, X. Waintal, What limits the simulation of quantum computers? *Phys. Rev. X* **10**, 041038 (2020).
23. J. Tindall, M. Fishman, E. M. Stoudenmire, D. Sels, Efficient tensor network simulation of IBM's Eagle kicked ising experiment. *PRX Quantum* **5**, 010308 (2024).
24. T. Begušić, J. Gray, G. K.-L. Chan, Fast and converged classical simulations of evidence for the utility of quantum computing before fault tolerance. *Sci. Adv.* **10**, eadk4321 (2024).
25. N. Gourianov, M. Lubasch, S. Dolgov, Q. Y. van den Berg, H. Babaee, P. Givi, M. Kiffner, D. Jaksch, A quantum-inspired approach to exploit turbulence structures. *Nat. Comput. Sci.* **2**, 30–37 (2022).
26. E. Ye, N. F. Loureiro, Quantum-inspired method for solving the Vlasov-Poisson equations. *Phys. Rev. E* **106**, 035208 (2022).
27. E. Ye, N. F. Loureiro, Quantized tensor networks for solving the Vlasov-Maxwell equations. arXiv:2311.07756 [physics.comp-ph] (2024).
28. R. D. Peddinti, S. Pisoni, A. Marini, P. Lott, H. Argentieri, E. Tiunov, L. Aolita, Quantum-inspired framework for computational fluid dynamics. *Commun. Phys.* **7**, 135 (2024).
29. L. Hölscher, P. Rao, L. Müller, J. Klepsch, A. Luckow, T. Stollenwerk, F. K. Wilhelm, Quantum-inspired fluid simulation of 2D turbulence with GPU acceleration. arXiv:2406.17823 [physics.flu-dyn] (2024).
30. M. Ritter, Y. Núñez Fernández, M. Wallerberger, J. von Delft, H. Shinaoka, X. Waintal, Quantics tensor cross interpolation for high-resolution parsimonious representations of multivariate functions. *Phys. Rev. Lett.* **132**, 056501 (2024).
31. S. Rohshap, M. K. Ritter, H. Shinaoka, J. von Delft, M. Wallerberger, A. Kauch, Two-particle calculations with quantics tensor trains – Solving the parquet equations. arXiv:2410.22975 [cond-mat.str-el] (2024).
32. F. A. Jaberi, R. S. Miller, C. K. Madnia, P. Givi, Non-Gaussian scalar statistics in homogeneous turbulence. *J. Fluid Mech.* **313**, 241–282 (1996).
33. J. H. Chen, Petascale direct numerical simulation of turbulent combustion–Fundamental insights toward predictive models. *Proc. Combust. Inst.* **33**, 99–123 (2011).
34. A. G. Nouri, M. B. Nik, P. Givi, D. Livescu, S. B. Pope, Self-contained filtered density function. *Phys. Rev. Fluids* **2**, 094603 (2017).
35. P. Givi, Filtered density function for subgrid scale modeling of turbulent combustion. *AIAA J.* **44**, 16–23 (2006).
36. J. Smagorinsky, General circulation experiments with the primitive equations. I. The basic experiment. *Monthly Weather Rev.* **91**, 99–164 (1963).
37. E. E. O'Brien, The probability density function (PDF) approach to reacting turbulent flows, in *Turbulent Reacting Flows*, P. A. Libby, F. A. Williams, Eds. (Springer-Verlag, Heidelberg), vol. 44 of *Topics in Applied Physics*, chap. 5, pp. 185–218 (1980).
38. M. Kiffner, D. Jaksch, Tensor network reduced order models for wall-bounded flows. *Phys. Rev. Fluids* **8**, 124101 (2023).
39. N. Gourianov, Exploiting *the structure of turbulence with tensor networks*, Ph.D. thesis, University of Oxford (2022).
40. M. Lubasch, P. Moinier, D. Jaksch, Multigrid renormalization. *J. Comput. Phys.* **372**, 587–602 (2018).
41. I. Oseledets, E. Tyrtyshnikov, TT-cross approximation for multidimensional arrays. *Linear Algebra Appl.* **432**, 70–88 (2010).
42. S. Dolgov, D. Savostyanov, Parallel cross interpolation for high-precision calculation of high-dimensional integrals. *Comput. Phys. Commun.* **246**, 106869 (2020).
43. B. Ghahremani, H. Babaee, A DEIM Tucker tensor cross algorithm and its application to dynamical low-rank approximation. *Comput. Methods Appl. Mech. Eng.* **423**, 116879 (2024).
44. Y. Núñez Fernández, M. K. Ritter, M. Jeannin, J.-W. Li, T. Kloss, T. Louvet, S. Terasaki, O. Parcollet, J. von Delft, H. Shinaoka, X. Waintal, Learning tensor networks with tensor cross interpolation: New algorithms and libraries. arXiv:2407.02454 [physics.comp-ph] (2024).
45. A. A. Michailidis, eC. Fenton, M. Kiffner, Tensor train multiplication. arXiv:2410.19747 [physics.comp-ph] (2024).
46. M. Ganahl, J. Beall, M. Hauru, A. G. M. Lewis, T. Wojno, J. H. Yoo, Y. Zou, G. Vidal, Density matrix renormalization group with tensor processing units. *PRX Quantum* **4**, 010317 (2023).
47. I. Glasser, R. Sweke, N. Pancotti, J. Eisert, J. I. Cirac, Expressive power of tensor-network factorizations for probabilistic modeling. *Adv. Neural Inf. Process. Syst.* **32**, 1496–1508 (2019).
48. F. Verstraete, M. M. Wolf, D. Perez-Garcia, J. I. Cirac, Criticality, the area law, and the computational power of projected entangled pair states. *Phys. Rev. Lett.* **96**, 220601 (2006).

49. V. Murg, F. Verstraete, O. Legeza, R. M. Noack, Simulating strongly correlated quantum systems with tree tensor networks. *Phys. Rev. B* **82**, 205105 (2010).
50. G. Evenbly, G. Vidal, Class of highly entangled many-body states that can be efficiently simulated. *Phys. Rev. Lett.* **112**, 240502 (2014).
51. D. Jaksch, P. Givi, A. J. Daley, T. Rung, Variational quantum algorithms for computational fluid dynamics. *AIAA Journal* **61**, 1885–1894 (2023).
52. R. Peng, J. Gray, G. K.-L. Chan, Arithmetic circuit tensor networks, multivariable function representation, and high-dimensional integration. *Phys. Rev. Res.* **5**, 013156 (2023).
53. A. Favre, H. Guitton, J. Guitton, A. Lichnerowicz, *Chaos and Determinism: Turbulence as a Paradigm for Complex Systems Converging Toward Final States* (Johns Hopkins Univ. Press) (1995).
54. D. Bandak, A. A. Mailybaev, G. L. Eyink, N. Goldenfeld, Spontaneous stochasticity amplifies even thermal noise to the largest scales of turbulence in a few eddy turnover times. *Phys. Rev. Lett.* **132**, 104002 (2024).
55. S. Holtz, T. Rohwedder, R. Schneider, On manifolds of tensors of fixed TT-rank. *Numer. Math.* **120**, 701–731 (2012).
56. J. Gray, quimb: A Python library for quantum information and many-body calculations. *J. Open Source Softw.* **3**, 819 (2018).

Acknowledgments: We thank A. Louis at the University of Oxford for discussions and would like to acknowledge the use of the University of Oxford Advanced Research Computing (ARC) facility in carrying out this work (http://dx.doi.org/10.5281/zenodo.22558). For the purpose of open access, a CC BY public copyright license is applied to this manuscript. **Funding:** N.G. acknowledges support by US-AFSOR grant FA8655-22-1-7027 and the UKRI "Quantum Computing and Simulation Hub" grant EP/P009565/1. P.G. acknowledges support by US-AFOSR grant FA9550-23-1-0014. D.J. acknowledges support by the European Union's Horizon Programme (HORIZON-CL42021-DIGITALEMERGING-02-10) grant agreement 101080085 QCFD, the Cluster of Excellence "Advanced Imaging of Matter" of the Deutsche Forschungsgemeinschaft (DFG), EXC 2056, project ID 390715994, and the Hamburg Quantum Computing Initiative (HQIC) project EFRE. The project is cofinanced by ERDF of the European Union and by "Fonds of the Hamburg Ministry of Science, Research, Equalities and Districts (BWFGB)". **Author contributions:** N.G. and P.G. conceived and planned the research project. N.G. formulated the MPS algorithm, did the analytical calculations, and wrote the software. The numerical experiments were jointly designed by all the authors and executed by N.G. The numerical results were analyzed and interpreted jointly by all the authors. N.G. and P.G. wrote the manuscript, with contributions from the other authors. The project was supervised by P.G. and S.B.P. **Competing interests:** The authors declare that they have no competing interests. **Data and materials availability:** All software and data accompanying this manuscript are publicly available at https://github.com/nikitn2/tendeq and https://doi.org/10.5281/zenodo.14223424. All data needed to evaluate the conclusions of the paper are present in the paper and/or the Supplementary Materials.

Submitted 20 August 2024
Accepted 27 December 2024
Published 29 January 2025
10.1126/sciadv.ads5990
