# Supplementary material for: Tensor networks enable the calculation of turbulence probability distributions
Source: Sci Adv. 2025 Jan 29;11(5):eads5990. doi: 10.1126/sciadv.ads5990 (PMC13109950; doi:10.1126/sciadv.ads5990)
Supplement: Supplementary file 2 — Supplementary Text Figs. S1 to S3 References [file sciadv.ads5990_sm.pdf]

Science Advances
AAAS

Supplementary Materials for

Tensor networks enable the calculation of turbulence probability distributions

Nikita Gourianov *et al.*

Corresponding author: Nikita Gourianov, nikgourianov@icloud.com

Sci. Adv. **11**, eads5990 (2025)
DOI: 10.1126/sciadv.ads5990

This PDF file includes:

Supplementary Text
Figs. S1 to S3
References

Supplementary Text

Separability of Fokker-Planck equation

Here we explain how the Main Text Fokker Planck Equation (2) becomes separable when $C_\Omega = 0$. Assume the PDF can be written in the form

$$f(\varphi_1, \varphi_2, x_1, x_2, x_3, t) = g(x_1, t)h(\varphi_1, t)R(\varphi_2, x_2, x_3, t). \tag{S1}$$

Inserting this into Equation (2), gives

$$\begin{aligned}
&hR\left(\frac{\partial g}{\partial t} + \langle U_1\rangle \frac{\partial g}{\partial x_1} - \frac{\partial}{\partial x_1}\left[(\gamma + \gamma_{\mathrm{SGS}})\frac{\partial g}{\partial x_1}\right]\right) + \\
&gR\left(\frac{\partial h}{\partial t} - \Omega_{\mathrm{mix}}\frac{\partial}{\partial \varphi_1}\left[(\varphi_1 - \langle \Phi_1\rangle)h\right] - C_r\varphi_2\frac{\partial}{\partial \varphi_1}[\varphi_1 h]\right) + \\
&gh\Bigg(\frac{\partial R}{\partial t} + \sum_{i=2,3}\left\{\langle U_i\rangle \frac{\partial R}{\partial x_i} - \frac{\partial}{\partial x_i}\left[(\gamma + \gamma_{\mathrm{SGS}})\frac{\partial R}{\partial x_i}\right]\right\} - \\
&\Omega_{\mathrm{mix}}\frac{\partial}{\partial \varphi_2}\left[(\varphi_2 - \langle \Phi_2\rangle)R\right] - C_r\varphi_1\frac{\partial}{\partial \varphi_2}[\varphi_2 R]\Bigg) = 0.
\end{aligned} \tag{S2}$$

Note here that it would be possible to solve for g, h, R separately if not for the nonlinear term $\langle \Phi_\alpha \rangle = \int \varphi_\alpha f \mathrm{d}\varphi_1 \mathrm{d}\varphi_2$. This term can however be eliminated from the above equation by setting $C_\Omega = 0 \rightarrow \Omega_{\mathrm{mix}} = 0$, thus completely decoupling the solutions along x_1, φ_1 from each other and the other dimensions. In fact, it is straightforward to show that setting $C_\Omega = 0$ will decouple *all* the dimensions from each other, turning Eq. (2) of the Main Text into a separable PDE with product-state solutions. Thus at low C_Ω, there should only be limited correlation between the different dimensions.

Empirical computational cost

The Main Text outlines the theoretical complexity of our algorithm. This section shows the empirical computational cost. The CPU-times referenced here are all measured on a *single* CPU core, by selecting the wall-time of the longest of the first 16 time-steps (out of the full M_t required to complete the time-evolution) of the algorithm (at $C_\Omega = 1$, Da = 0).

Single-core times as functions of χ, M are shown in Figs. S1 and S2. The first figure shows that at small χ, the cost of the program is dominated by the overhead of the Python interpreter, while at

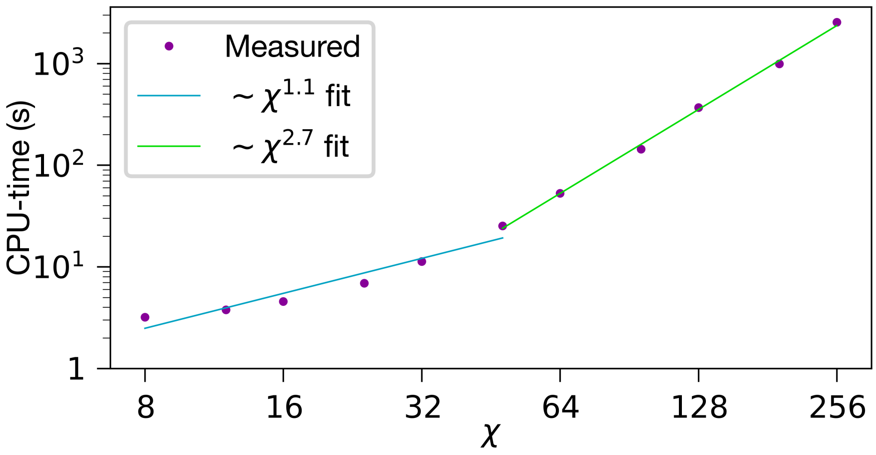

Figure S1: **Empirical cost of MPS-TN algorithm**. The single-core CPU-time (in seconds) required for a typical time-step of the MPS algorithm is shown for different χ. The algorithm was implemented in Python 3 [on top of the quimb library (*56*)] and run on a *single* core from a Intel Xeon 8268 CPU.

intermediate χ, the $\sim \chi^4$ element-wise products are too few to practically dominate the many $\sim \chi^2$ and $\sim \chi^3$ operations (outlined in the Main Text) of the algorithm. Fig. S2 empirically confirms the $\sim \log M$ scaling of the MPS algorithm, in contrast to the $\sim M^d$ cost required by an equivalent, standard FD scheme. Note how the MPS algorithm at $\chi = 32, M = 128$ requires $O(1/10^3)$ of the flops of the equivalent FD scheme.

Main Text Figs. 2b, 2c can be combined with Fig. S1 to show how the observed accuracy of the MPS algorithm increases with computational effort. This relationship is illustrated in Supplementary Fig. S3.

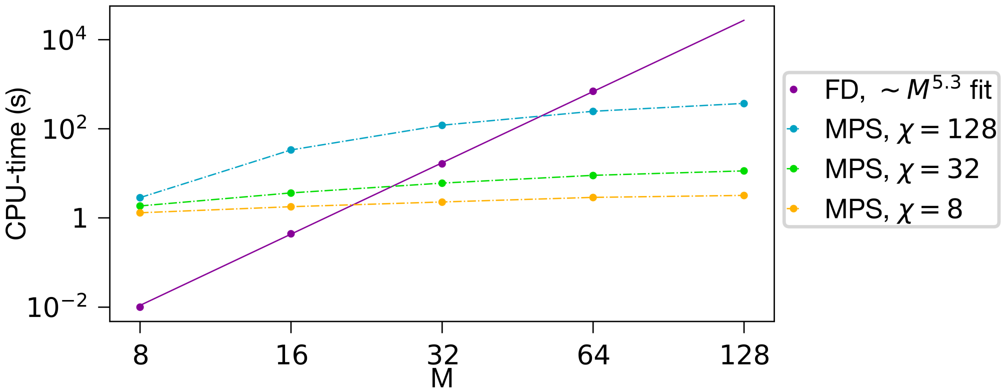

Figure S2: **MPS-TN versus FD scheme empirical cost**. Equivalent single-core CPU-time to Fig. S1, except now plotted across M for both the MPS algorithm at various χ and an equivalent, standard FD scheme (implemented in Python 3). For the FD scheme, the $M = 128$ datapoint could not be measured due to the extreme memory load required.

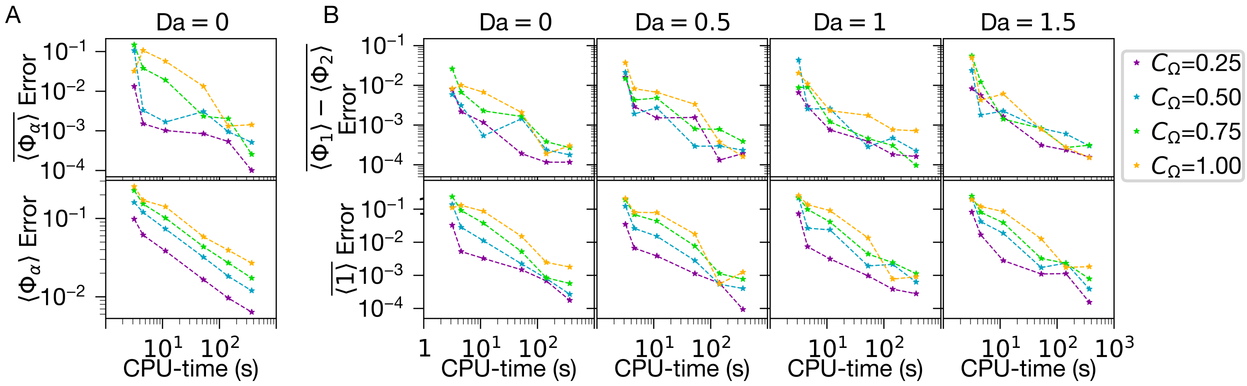

Figure S3: **Accuracy convergence of MPS-TN algorithm versus empirical computational cost**. Here the errors of Main Text Fig. 2 are plotted as functions of the single-core CPU time measured in Fig. S1. **A** corresponds to the Fig. 2b errors, and **B** to the Fig. 2c ones.

REFERENCES AND NOTES

1. E. Hopf, Statistical hydromechanics and functional calculus. *J. Rat. Mech. Anal.* **1**, 87–123 (1952).

2. A. S. Monin, A. M. Yaglom, *Statistical Fluid Mechanics* (MIT Press, Cambridge, MA) (1975).

3. S. B. Pope, *Turbulent Flows* (Cambridge Univ. Press, Cambridge, UK) (2012).

4. R. O. Fox, *Computational Models for Turbulent Reacting Flows* (Cambridge Univ. Press, Cambridge, UK) (2009).

5. C. Dopazo, Recent Developments in PDF Methods, in *Turbulent Reacting Flows*, P. A. Libby, F. A. Williams, Eds. (Academic Press, London, England), chap. 7, pp. 375–474 (1994).

6. F. A. Williams, *Combustion Theory* (The Benjamin/Cummings Publishing Company, Menlo Park, CA), Ed. 2. (1985).

7. D. Livescu, A. G. Nouri, F. Battaglia, P. Givi, Eds. *Modeling and Simulation of Turbulent Mixing and Reaction: For Power, Energy and Flight* (Springer, Germany) (2020).

8. S. B. Pope, PDF methods for turbulent reactive flows. *Prog. Energy Combust. Sci.* **11**, 119–192 (1985).

9. V. Hiremath, S. R. Lantz, H. Wang, S. B. Pope, Computationally-efficient and scalable parallel implementation of chemistry in simulations of turbulent combustion. *Combust. Flame* **159**, 3096–3109 (2012).

10. H. Zhou, P. Givi, Z. Ren, Filtered density function: A stochastic closure for coarse grained simulation, in *Coarse Graining Turbulence: Modeling and Data-Driven Approaches and their Applications* (Cambridge Univ. Press), chap. 4 (2025).

11. D. Poulin, A. Qarry, R. Somma, F. Verstraete, Quantum simulation of time-dependent Hamiltonians and the convenient illusion of Hilbert space. *Phys. Rev. Lett.* **106**, 170501 (2011).

12. S. R. White, Density matrix formulation for quantum renormalization groups. *Phys. Rev. Lett.* **69**, 2863–2866 (1992).

13. G. Vidal, Efficient classical simulation of slightly entangled quantum computations. *Phys. Rev. Lett.* **91**, 147902 (2003).

14. U. Schollwöck, The density-matrix renormalization group in the age of matrix product states. *Ann. Phys.* **326**, 96–192 (2011).

15. R. Orús, Tensor networks for complex quantum systems. *Nat. Rev. Phys.* **1**, 538–550 (2019).

16. S. R. Clark, D. Jaksch, Dynamics of the superfluid to Mott-insulator transition in one dimension. *Phys. Rev. A* **70**, 043612 (2004).

17. A. Feiguin, S. Trebst, A. W. W. Ludwig, M. Troyer, A. Kitaev, Z. Wang, M. H. Freedman, Interacting anyons in topological quantum liquids: The golden chain. *Phys. Rev. Lett.* **98**, 160409 (2007).

18. M. Cheneau, P. Barmettler, D. Poletti, M. Endres, P. Schauß, T. Fukuhara, C. Gross, I. Bloch, C. Kollath, S. Kuhr, Light-cone-like spreading of correlations in a quantum many-body system. *Nature* **481**, 484–487 (2012).

19. S. Trotzky, Y.-A. Chen, A. Flesch, I. P. McCulloch, U. Schollwöck, J. Eisert, I. Bloch, Probing the relaxation towards equilibrium in an isolated strongly correlated one-dimensional Bose gas. *Nat. Phys.* **8**, 325–330 (2012).

20. B.-X. Zheng, C.-M. Chung, P. Corboz, G. Ehlers, M.-P. Qin, R. M. Noack, H. Shi, S. R. White, S. Zhang, G. K.-L. Chan, Stripe order in the underdoped region of the two-dimensional Hubbard model. *Science* **358**, 1155–1160 (2017).

21. C. Huang, F. Zhang, M. Newman, X. Ni, D. Ding, J. Cai, X. Gao, T. Wang, F. Wu, G. Zhang, H.-S. Ku, Z. Tian, J. Wu, H. Xu, H. Yu, B. Yuan, M. Szegedy, Y. Shi, H.-H. Zhao, C. Deng, J. Chen, Efficient parallelization of tensor network contraction for simulating quantum computation. *Nat. Comput. Sci.* **1**, 578–587 (2021).

22. Y. Zhou, E. M. Stoudenmire, X. Waintal, What limits the simulation of quantum computers? *Phys. Rev. X* **10**, 041038 (2020).

23. J. Tindall, M. Fishman, E. M. Stoudenmire, D. Sels, Efficient tensor network simulation of IBM's Eagle kicked ising experiment. *PRX Quantum* **5**, 010308 (2024).

24. T. Begušić, J. Gray, G. K.-L. Chan, Fast and converged classical simulations of evidence for the utility of quantum computing before fault tolerance. *Sci. Adv.* **10**, eadk4321 (2024).

25. N. Gourianov, M. Lubasch, S. Dolgov, Q. Y. van den Berg, H. Babaee, P. Givi, M. Kiffner, D. Jaksch, A quantum-inspired approach to exploit turbulence structures. *Nat. Comput. Sci.* **2**, 30–37 (2022).

26. E. Ye, N. F. Loureiro, Quantum-inspired method for solving the Vlasov-Poisson equations. *Phys. Rev. E* **106**, 035208 (2022).

27. E. Ye, N. F. Loureiro, Quantized tensor networks for solving the Vlasov-Maxwell equations. arXiv:2311.07756 [physics.comp-ph] (2024).

28. R. D. Peddinti, S. Pisoni, A. Marini, P. Lott, H. Argentieri, E. Tiunov, L. Aolita, Quantum-inspired framework for computational fluid dynamics. *Commun. Phys.* **7**, 135 (2024).

29. L. Hölscher, P. Rao, L. Müller, J. Klepsch, A. Luckow, T. Stollenwerk, F. K. Wilhelm, Quantum-inspired fluid simulation of 2D turbulence with GPU acceleration. arXiv:2406.17823 [physics.flu-dyn] (2024).

30. M. Ritter, Y. Núñez Fernández, M. Wallerberger, J. von Delft, H. Shinaoka, X. Waintal, Quantics tensor cross interpolation for high-resolution parsimonious representations of multivariate functions. *Phys. Rev. Lett.* **132**, 056501 (2024).

31. S. Rohshap, M. K. Ritter, H. Shinaoka, J. von Delft, M. Wallerberger, A. Kauch, Two-particle calculations with quantics tensor trains – Solving the parquet equations. arXiv:2410.22975 [cond-mat.str-el] (2024).

32. F. A. Jaberi, R. S. Miller, C. K. Madnia, P. Givi, Non-Gaussian scalar statistics in homogeneous turbulence. *J. Fluid Mech.* **313**, 241–282 (1996).

33. J. H. Chen, Petascale direct numerical simulation of turbulent combustion–Fundamental insights toward predictive models. *Proc. Combust. Inst.* **33**, 99–123 (2011).

34. A. G. Nouri, M. B. Nik, P. Givi, D. Livescu, S. B. Pope, Self-contained filtered density function. *Phys. Rev. Fluids* **2**, 094603 (2017).

35. P. Givi, Filtered density function for subgrid scale modeling of turbulent combustion. *AIAA J.* **44**, 16–23 (2006).

36. J. Smagorinsky, General circulation experiments with the primitive equations. I. The basic experiment. *Monthly Weather Rev.* **91**, 99–164 (1963).

37. E. E. O'Brien, The probability density function (PDF) approach to reacting turbulent flows, in *Turbulent Reacting Flows*, P. A. Libby, F. A. Williams, Eds. (Springer-Verlag, Heidelberg), vol. 44 of *Topics in Applied Physics*, chap. 5, pp. 185–218 (1980).

38. M. Kiffner, D. Jaksch, Tensor network reduced order models for wall-bounded flows. *Phys. Rev. Fluids* **8**, 124101 (2023).

39. N. Gourianov, Exploiting *the structure of turbulence with tensor networks*, Ph.D. thesis, University of Oxford (2022).

40. M. Lubasch, P. Moinier, D. Jaksch, Multigrid renormalization. *J. Comput. Phys.* **372**, 587–602 (2018).

41. I. Oseledets, E. Tyrtyshnikov, TT-cross approximation for multidimensional arrays. *Linear Algebra Appl.* **432**, 70–88 (2010).

42. S. Dolgov, D. Savostyanov, Parallel cross interpolation for high-precision calculation of high-dimensional integrals. *Comput. Phys. Commun.* **246**, 106869 (2020).

43. B. Ghahremani, H. Babaee, A DEIM Tucker tensor cross algorithm and its application to dynamical low-rank approximation. *Comput. Methods Appl. Mech. Eng.* **423**, 116879 (2024).

44. Y. Núñez Fernández, M. K. Ritter, M. Jeannin, J.-W. Li, T. Kloss, T. Louvet, S. Terasaki, O. Parcollet, J. von Delft, H. Shinaoka, X. Waintal, Learning tensor networks with tensor cross interpolation: New algorithms and libraries. arXiv:2407.02454 [physics.comp-ph] (2024).

45. A. A. Michailidis, eC. Fenton, M. Kiffner, Tensor train multiplication. arXiv:2410.19747 [physics.comp-ph] (2024).

46. M. Ganahl, J. Beall, M. Hauru, A. G. M. Lewis, T. Wojno, J. H. Yoo, Y. Zou, G. Vidal, Density matrix renormalization group with tensor processing units. *PRX Quantum* **4**, 010317 (2023).

47. I. Glasser, R. Sweke, N. Pancotti, J. Eisert, J. I. Cirac, Expressive power of tensor-network factorizations for probabilistic modeling. *Adv. Neural Inf. Process. Syst.* **32**, 1496–1508 (2019).

48. F. Verstraete, M. M. Wolf, D. Perez-Garcia, J. I. Cirac, Criticality, the area law, and the computational power of projected entangled pair states. *Phys. Rev. Lett.* **96**, 220601 (2006).

49. V. Murg, F. Verstraete, O. Legeza, R. M. Noack, Simulating strongly correlated quantum systems with tree tensor networks. *Phys. Rev. B* **82**, 205105 (2010).

50. G. Evenbly, G. Vidal, Class of highly entangled many-body states that can be efficiently simulated. *Phys. Rev. Lett.* **112**, 240502 (2014).

51. D. Jaksch, P. Givi, A. J. Daley, T. Rung, Variational quantum algorithms for computational fluid dynamics. *AIAA Journal* **61**, 1885–1894 (2023).

52. R. Peng, J. Gray, G. K.-L. Chan, Arithmetic circuit tensor networks, multivariable function representation, and high-dimensional integration. *Phys. Rev. Res.* **5**, 013156 (2023).

53. A. Favre, H. Guitton, J. Guitton, A. Lichnerowicz, *Chaos and Determinism: Turbulence as a Paradigm for Complex Systems Converging Toward Final States* (Johns Hopkins Univ. Press) (1995).

54. D. Bandak, A. A. Mailybaev, G. L. Eyink, N. Goldenfeld, Spontaneous stochasticity amplifies even thermal noise to the largest scales of turbulence in a few eddy turnover times. *Phys. Rev. Lett.* **132**, 104002 (2024).

55. S. Holtz, T. Rohwedder, R. Schneider, On manifolds of tensors of fixed TT-rank. *Numer. Math.* **120**, 701–731 (2012).

56. J. Gray, quimb: A Python library for quantum information and many-body calculations. *J. Open Source Softw.* **3**, 819 (2018).
